# Prognostic impact of resting full-cycle ratio and diastolic non-hyperemic pressure ratios in patients with deferred revascularization

**DOI:** 10.1007/s00392-022-02149-1

**Published:** 2023-01-05

**Authors:** Marcel Halbach, Christopher Ameskamp, Victor Mauri, Angela Ernst, Philipp Lake, Stephan Nienaber, Stephan Baldus, Matti Adam, Hendrik Wienemann

**Affiliations:** 1 Clinic III for Internal Medicine, University of Cologne, Faculty of Medicine and University Hospital Cologne, Kerpener Str. 61, 50937 Cologne, Germany; 2 Institute of Medical Statistics and Computational Biology, University of Cologne, Robert-Koch-Str. 10, 50931 Cologne, Germany

**Keywords:** Coronary artery disease, Fractional flow reserve, Invasive coronary angiography, Resting full-cycle ratio

## Abstract

**Background:**

Non-hyperemic pressure ratios (NHPRs) like resting full-cycle ratio (RFR), diastolic pressure ratio during entire diastole (dPR[entire]) and diastolic pressure ratio during wave-free period (dPR[WFP]) are increasingly used to guide revascularization. The effect of NHPRs on mid-term prognosis has not been well established.

**Objective:**

We investigated the prognostic implications of NHRPs in patients whose revascularization was deferred based on fractional flow reserve (FFR) in a single-centre population.

**Methods:**

NHPRs and FFR were calculated offline from pressure tracings by an independent core laboratory. Follow-up data were acquired through records of hospital visits or telephone interviews. The primary outcome was a vessel-oriented composite outcome (VOCO) (a composite of cardiac death, vessel-related myocardial infarction, and ischemia-driven revascularization) in deferred vessels at 2 years.

**Results:**

316 patients with 377 deferred lesions were analysed. Discordance of NHPRs and FFR was found in 13.0–18.3% of lesions. The correlation coefficient between NHPRs was 0.99 (95% confidence interval 0.99–1.00). At 2 years, VOCO occurred in 19 lesions (5.0%). Estimated glomerular filtration rate < 30 mL/min/1.73 m^2^ [hazard ratio (HR) 5.7, *p* = 0.002], previous myocardial infarction (HR 3.3, *p* = 0.018), diabetes (HR 2.7, *p* = 0.042), RFR ≤ 0.89 (HR 2.7, *p* = 0.041) and dPR[WFP] ≤ 0.89 (HR 2.7, *p* = 0.049) were associated with higher incidence of VOCO at 2 years in the univariable analysis. A non-significant trend was found for dPR[entire] (HR 1.9, *p* = 0.26).

**Conclusion:**

A positive RFR or dPR[WFP] were associated with a worse prognosis in deferred lesions, suggesting that the use of NHPRs in addition to FFR may improve risk estimation.

**Graphical abstract:**

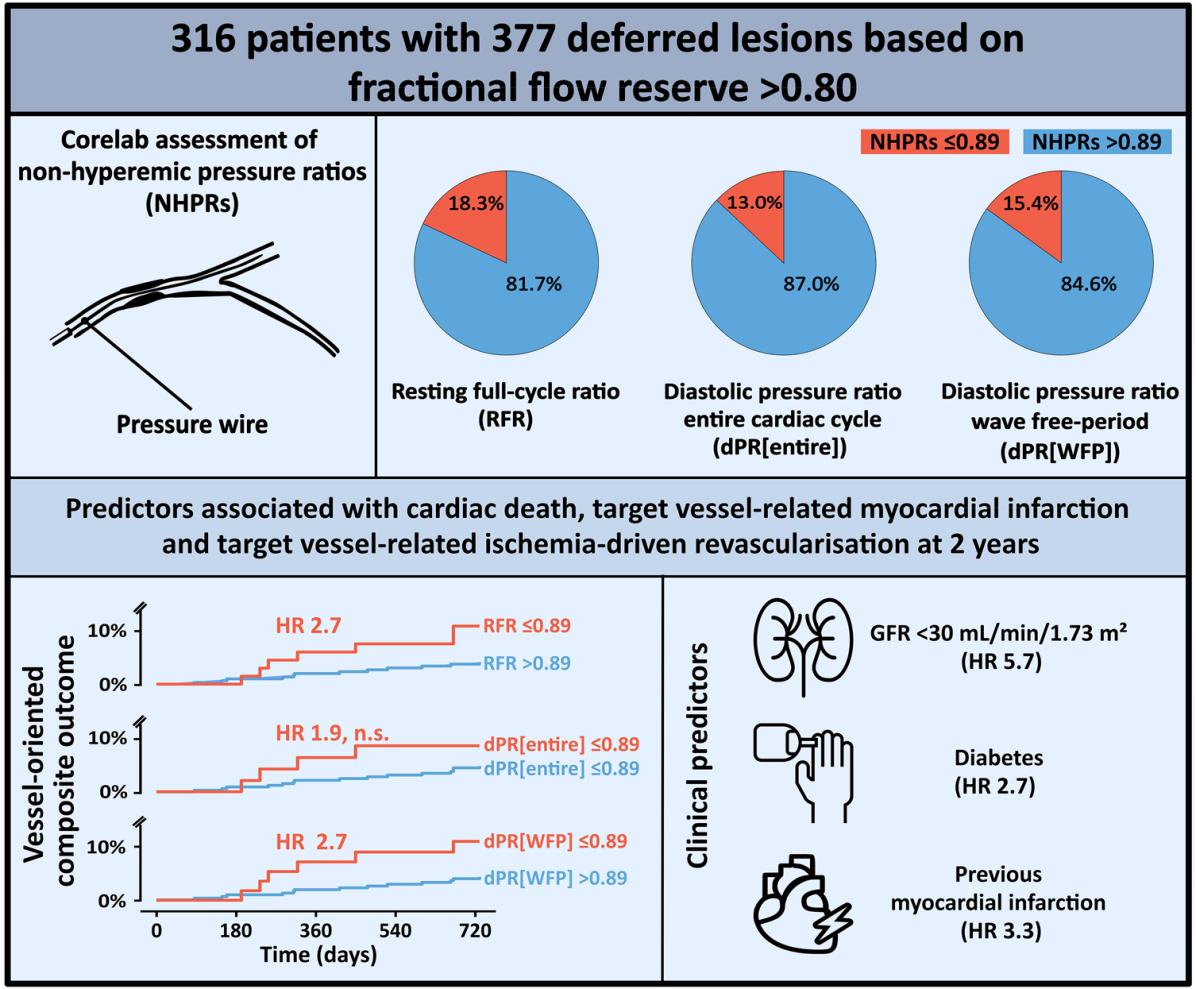

**Supplementary Information:**

The online version contains supplementary material available at 10.1007/s00392-022-02149-1.

## Introduction

Physiological assessment of intermediate coronary stenoses using fractional flow reserve (FFR) reduces the number of implanted stents and may avoid major adverse cardiac events compared to an angiography-based approach [1, 2]. However, adoption of this technology in daily practice is limited by adenosine usage, which prolongs examination time and increases costs. The easier use of adenosine-free non-hyperemic resting indices (NHPRs) has expanded the applicability of invasive physiological assessments. Several NHPRs have been proposed including diastolic pressure ratios like dPR[entire] [also referred to as dPR; mean distal coronary pressure to aortic pressure ratio (Pd/Pa) during the entire diastole] and dPR[WFP] (mean Pd/Pa during the wave-free period of diastole, equivalent to the commercially available instantaneous wave-free ratio (iFR) [3, 4]), or the resting full-cycle ratio (RFR), which is the lowest Pd/Pa over systole and diastole [5]. While NHPRs have an excellent correlation with each other, discordance between FFR and NHPRs are reported in up to 22% of all cases [6, 7].

Two large randomized studies demonstrated the non-inferiority of an iFR-guided strategy compared to an FFR-guided strategy for a follow-up of 12 months and 5 years [8–10], despite differences in revascularization rate, leading to a class I guideline recommendation to use either FFR or iFR to assess the hemodynamic relevance of intermediate stenoses [11]. Randomized studies on other NHPRs are still lacking and outcome data from non-randomized studies are sparse. Several publications by Lee et al. reported clinical outcome after measurements of NHPRs in an Asian population with several exclusion criteria, which may not be representative for real-life patients in western countries [12–14].

The purpose of the current study was to investigate the prognostic impact of RFR, dPR[WFP] and dPR[entire] in a real-life cohort with deferred lesions based on FFR measurements.

## Methods

### Patient population

We examined patients with intermediate coronary stenosis (visual grading between 30 and 80%) and pressure wire assessment at our centre over a 4-year period with the last patient included in February 2019. Pressure wire recordings were not performed in the following settings: (1) contraindication for adenosine, (2) cardiogenic shock, (3) ST-segment elevation myocardial infarction, (4) culprit vessels in the setting of myocardial infarction, (5) stenosis technically not suitable for analysis and (6) lesions without myocardial viability. Two hundred and sixty-six patients with 296 lesions were excluded from the analysis due to revascularization at the index measurement and lack of a deferred lesion. Patients with non-revascularized vessels despite FFR ≤ 0.80 and functional assessment of a coronary artery bypass graft or left main artery were also excluded from the analysis.

The local ethics committee approved the study. Part of the study was registered at the German Clinical Trial Register (DRKS-Study-ID: DRKS00022842; https://www.drks.de/drks_web).

### Quantitative coronary angiography

Quantitative coronary angiography was performed to measure diameter stenosis, minimum luminal diameter, reference-vessel size, and lesion length with the validated software Medical CAAS II (Pie Medical Imaging, Maastricht, The Netherlands) or Medis (Medical Imaging System, Leiden, The Netherlands).

### Acquisition and analysis of FFR and NHPRs

Interventional procedures and medication were applied according to current guidelines and local practice by using a transfemoral or transradial approach. A pressure guide wire [Pressure Wire™ X Guidewire (Abbott Vascular Inc., Santa Clara, CA)] was positioned at the distal segment of the target vessel, and intracoronary nitroglycerine was given before pressure wire recordings. Maximal hyperemia was induced by infusion of adenosine through a forearm vein at 140 μg/kg/min. At the end of the procedure the pressure drift was checked with the pressure sensor at the level of the coronary ostium.

Pressure traces were anonymously transferred to an independent core laboratory (CoroLab; Coroventis Research AB, Uppsala, Sweden) for offline calculations of FFR, RFR, dPR[entire], dPR[WFP]. FFR was calculated as mean Pd/Pa at maximal hyperemia induced by infusion of adenosine (at least three cardiac cycles). Traces at baseline, i.e. before application of adenosine, were used to determine NHPRs. RFR was defined as the mean lowest Pd/Pa value irrespective of the cardiac cycle phase (i.e. in systole or diastole) during five cardiac cycles. dPR[WFP] represents the average Pd/Pa during the wave-free period (from 25% of the entire diastole to 5 ms before the end of diastole) in five cardiac cycles, according to the definition of Sen et al. [4]. dPR[entire] displays the average Pd/Pa over the entire diastole during five cardiac cycles. RFR ≤ 0.89, dPR[WFP] ≤ 0.89 and dPR[entire] ≤ 0.89 were considered significant, i.e. suggestive of myocardial ischemia in the interrogated vessel.

### Outcome

Follow-up data were acquired through local records of subsequent inpatient or outpatient hospital visits or by telephone interviews, if patients had no subsequent visits in our hospital covering the follow-up period. If patients or relatives reported external hospital submissions during the follow-up period, external reports were gathered. Nine patients with ten lesions were lost to follow-up.

The endpoint of this study was a vessel-oriented composite outcome (VOCO), consisting of cardiovascular death, target vessel-related myocardial infarction (diagnoses of myocardial infarction were derived from internal and external reports; during the study period, the third and fourth universal definition of myocardial infarction were applicable, i.e. myocardial infarction (type 1) defined as rise and/or fall of cardiac biomarkers together with the evidence of myocardial ischemia (symptoms, ECG changes, imaging evidence) [15, 16]), and target vessel-related ischemia-driven revascularization assessed at 2 years. When the cause of death was unknown or undeterminable, it was assumed to be of cardiac origin. Cut-off values of ≤ 0.89 for NHPRs were used to compare clinical outcomes according to positive or negative pressure-derived indices.

### Statistical analysis

Continuous variables are presented as median with interquartile range (IQR) from the 25th to 75th percentiles. Normal distribution was tested with QQ-plots and the Shapiro-Wilk test. Continuous variables were compared using the Wilcoxon rank sum test. Categorical variables are shown as frequencies and percentages and compared appropriately using Pearson’s Chi-square test or Fisher's exact test. Correlation between the physiological indices was assessed using Spearman’s coefficient. Data were examined on a per-patient level for clinical baseline characteristics. Lesion characteristics were examined on a per-vessel basis.

Survival curves and cumulative proportions were estimated using the Kaplan–Meier method and log-rank tests. Marginal cox proportional-hazards models were used to adjust the clustering of multiple vessel measurements in the same patient. Results are reported as hazard ratios (HR) and 95% confidence interval (CI). The proportional hazard assumption was confirmed by examination of curves and by testing of partial (Schoenfeld) residuals, and no relevant violations were found. A univariable analysis was performed on relevant clinical and angiographic data to determine predictors of VOCO. All variables that showed *p* values < 0.10 in simple cox regression were included in a multivariable cox regression model. Significance was defined as two-sided *p* value < 0.05. Statistical analyses were performed using SPSS Statistics (version 27; IBM, Armonk, New York) and R Statistical Software (version 4.2.1; R Foundation for Statistical Computing, Vienna, Austria).

## Results

### Baseline characteristics of patients

The total cohort was composed of 316 patients with 377 deferred lesions. The median age was 71 years (62.8, 78.0), and 34.8% were female. The population had typical risk factors of coronary artery disease patients with a high incidence of hypertension, hypercholesterolemia, and diabetes mellitus. 18.4% had acute coronary syndrome as clinical presentation (Table 1). 30.7% had a previous myocardial infarction. The baseline clinical characteristics of the whole cohort and RFR > 0.89 and RFR ≤ 0.89 subgroups are displayed in Table 1. Patients with RFR ≤ 0.89 were significantly older, suffered more often from peripheral artery disease and had a slightly worse glomerular filtration rate compared to patients with RFR > 0.89. Supplemental Tables 1a and 1b show the baseline characteristics for subgroups with positive and negative dPR[entire] and dPR[WFP].

**Table 1:** Baseline patient characteristics according to resting full-cycle ratio (RFR)

	Overall, *N* = 316	RFR > 0.89, *N* = 256	RFR ≤ 0.89, *N* = 60	*p* value
Female	110 (34.8%)	85 (33.2%)	25 (41.7%)	0.22
Age (years)	71.0 (62.8–78.0)	70.0 (62.0–76.0)	76.0 (67.8–81.0)	< 0.001
Body-mass-index (kg/m^2^)	27 (24.4–30.5)	27.5 (24.5–30.5)	26.5 (23.7–29.5)	0.10
Diabetes	82 (25.9%)	68 (26.6%)	14 (23.3%)	0.61
Hypertension	226 (71.5%)	182 (71.1%)	44 (73.3%)	0.73
Dyslipidemia	156 (49.4%)	123 (48.0%)	33 (55.0%)	0.33
Atrial fibrillation	42 (13.3%)	32 (12.5%)	10 (16.7%)	0.39
Former or current smoker	102 (32.3%)	78 (30.5%)	24 (40.0%)	0.16
Peripheral artery disease	23 (7.3%)	14 (5.5%)	9 (15.0%)	0.022
Previous stroke	34 (10.8%)	23 (9.0%)	11 (18.3%)	0.035
Family history of coronary artery disease	40 (12.7%)	30 (11.7%)	10 (16.7%)	0.30
eGFR (ml/min/1.73 m^2^)	61.6 (47.3–78.4)	63.2 (49.0–80.1)	51.0 (39.8–73.8)	0.023
Chronic kidney disease^a^	20 (6.3%)	14 (5.5%)	6 (10.0%)	0.23
Previous coronary artery bypass graft	23 (7.3%)	19 (7.4%)	4 (6.7%)	> 0.99
Previous myocardial infarction	97 (30.7%)	75 (29.3%)	22 (36.7%)	0.27
Clinical presentation				0.46
Acute coronary syndrome	58 (18.4%)	49 (19.1%)	9 (15.0%)	
Stable coronary artery disease	258 (81.6%)	207 (80.9%)	51 (85.0%)	
Lipid-lowering drugs	286 (90.5%)	232 (90.6%)	54 (90.0%)	0.88

Values are expressed as median (IQR) or *n* (%)

*eGFR* estimated glomerular filtration rate

^a^Estimated glomerular filtration rate (< 30 ml/min/1.73 m^2^)

### Vessel characteristics

The analysed vessels had a minimum lumen diameter of 1.51 (1.28–1.79) mm within the stenotic region, a reference diameter of 3.01 (2.64–3.42) mm, an obstruction length of 11.70 (8.28–17.59) mm and a per cent diameter stenosis of 50.0 (47.0–54.0)% (Table 2). 51.5% of lesions were in the left anterior descending coronary artery (LAD). Table 2 shows baseline lesion characteristics for subgroups with positive and negative RFR, Supplemental Tables 2a and 2b for dPR[entire] and dPR[WFP].

**Table 2:** Lesion characteristics according to resting full-cycle ratio (RFR)

	Overall, *N* = 377	RFR > 0.89, *N* = 308	RFR ≤ 0.89, *N* = 69	*p* value
Location of lesions				
Left anterior descending artery	194 (51.5%)	139 (45.1%)	55 (79.7%)	< 0.001
Circumflex artery	77 (20.4%)	72 (23.4%)	5 (7.2%)	0.003
Right coronary artery	100 (26.5%)	92 (29.9%)	8 (11.6%)	0.002
Ramus intermedius	6 (1.6%)	5 (1.6%)	1 (1.4%)	> 0.99
Proximal lesion	174 (46.2%)	146 (47.4%)	28 (40.6%)	0.30
Reference diameter (mm)	3.01 (2.64–3.42)	3.04 (2.70–3.44)	2.87 (2.45–3.21)	0.009
Minimum lumen diameter (mm)	1.51 (1.28–1.79)	1.53 (1.32–1.81)	1.41 (1.16–1.67)	0.003
Diameter stenosis (%)	50.0 (47.0–54.0)	50.0 (47.0–53.0)	52.0 (48.0–55.0)	0.023
Lesion length (mm)	11.70 (8.28–17.59)	11.86 (8.32–17.68)	10.70 (8.18–16.99)	0.64
Fractional flow reserve	0.88 (0.85–0.92)	0.89 (0.86–0.93)	0.84 (0.83–0.86)	< 0.001
Resting full-cycle ratio	0.94 (0.91–0.97)	0.95 (0.93–0.98)	0.88 (0.86–0.89)	< 0.001
dPR[entire]	0.95 (0.91–0.98)	0.96 (0.93–0.98)	0.89 (0.87–0.90)	< 0.001
dPR[WFP]	0.95 (0.91–0.98)	0.96 (0.93–0.98)	0.88 (0.87–0.89)	< 0.001

Values are expressed as median (IQR) or *n* (%)

*dPR[entire]* diastolic pressure ratio during entire diastole, *dPR[WFP]* diastolic pressure ratio during the wave-free period of the diastole

### Correlation and agreement between FFR and NHPRS

**Fig. 1:**
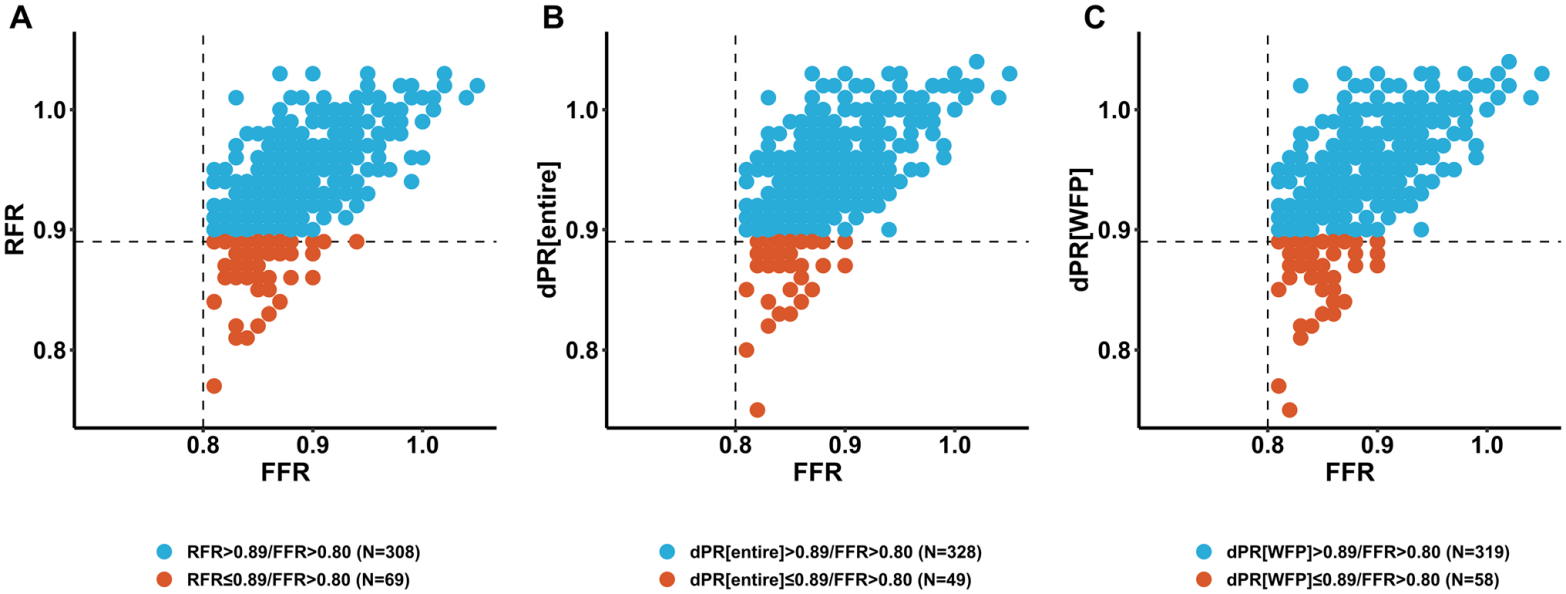
Distribution of RFR, dPR[WFP] and dPR[entire] compared to FFR. Please note that lesions with FFR ≤ 0.80 were excluded from the analysis. 69 lesions (18.3%) showed discordant results of FFR and RFR (**a**), 49 lesions (13.0%) showed discordant results of FFR and dPR[entire] (**b**) and 58 lesions (15.4%) showed discordant results of FFR and dPR[WFP] (**c**). *dPR[entire]* diastolic pressure ratio over the entire diastole, *dPR[WFP]* diastolic pressure ratio over the wave-free period, *FFR* fractional flow reserve, *RFR* resting full-cycle ratio

**Fig. 2:**
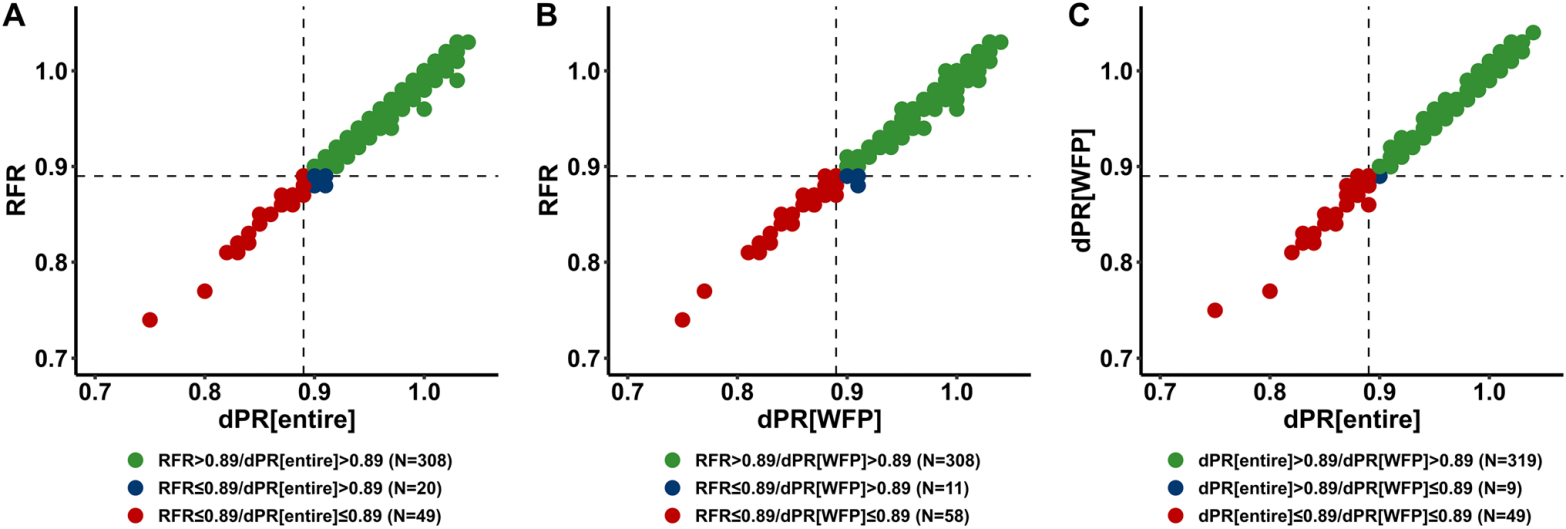
Correlation of RFR, dPR[WFP] and dPR[entire]. **a** RFR and dPR[entire], **b** dPR[entire] and dPR[WFP], **c** RFR and dPR[WFP]. *dPR[entire]* diastolic pressure ratio over the entire diastole, *dPR[WFP]* diastolic pressure ratio over the wave-free period, *RFR* resting full-cycle ratio

The correlation and frequency of NHPRs ≤ 0.89 and NHPRs > 0.89 for RFR, dPR[entire] and dPR[WFP] are shown in Fig. 1. A similar correlation coefficient was seen across all vessels between NHPRs and FFR; FFR was significantly correlated with RFR (*r* = 0.64, 95% CI 0.57–0.69, *p* < 0.001), dPR[entire] (*r* = 0.65, 95% CI 0.59–0.70, *p* < 0.001) and dPR[WFP] (*r* = 0.65, 95% CI 0.55–0.70, *p* < 0.001). 69 lesions (18.3%) showed discordant results of FFR and RFR, 49 lesions (13.0%) showed discordant results of FFR and dPR[entire] and 58 lesions (15.4%) showed discordant results of FFR and dPR[WFP]. The correlation of NHPRs among each other is displayed in Fig. 2 (*r* = 0.99, 95% CI 0.99–1.00 for RFR vs dPR[entire], *r* = 0.99, 95% CI 0.98–0.99 for RFR vs dPR[WFP], *r* = 0.99, 95% CI 0.99–1.00 for dPR[WFP] vs dPR[entire], for all *p* < 0.001).

## Clinical outcomes

**Fig. 3:**
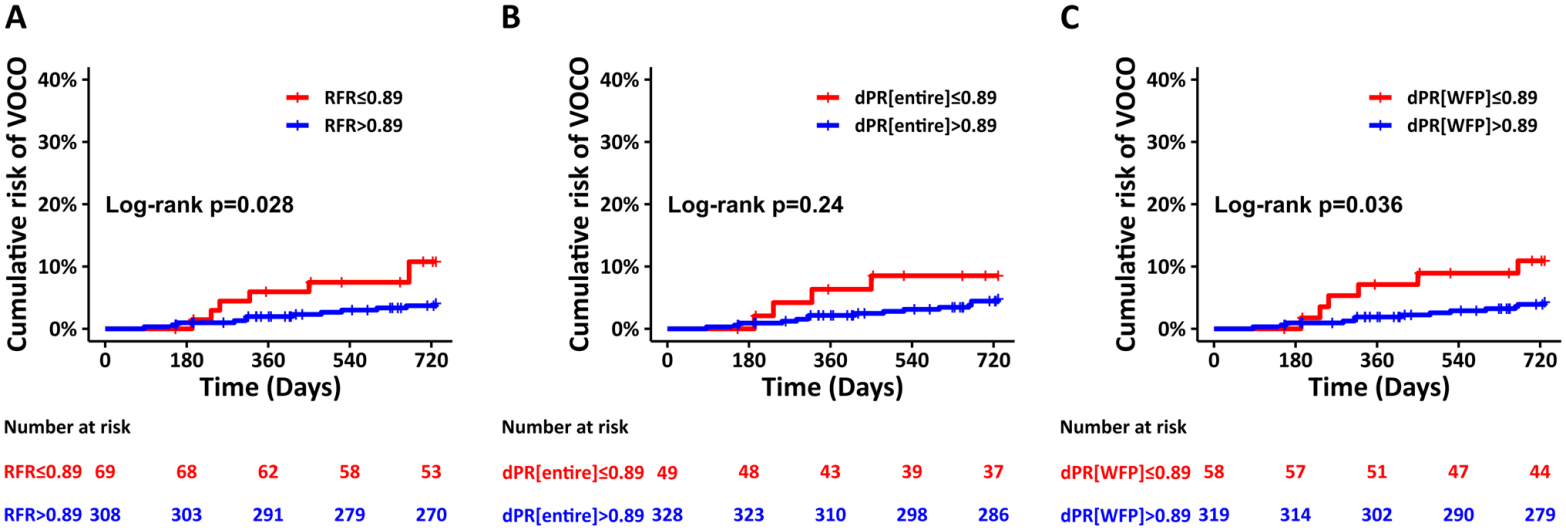
Kaplan–Meier curves of VOCO for RFR, dPR[WFP] and dPR[entire]. NHPRs ≤ 0.89 were associated with a worse outcome compared to NHPRs > 0.89 in deferred lesions (with deferral based on FFR > 0.80; **a**–**c**), this finding was statistically significant for RFR and dPR[WFP], with a similar trend without reaching statistical significance for dPR[entire]. *dPR[entire]* diastolic pressure ratio over the entire diastole, *dPR[WFP]* diastolic pressure ratio over the wave-free period, *NHPRs* non-hyperemic pressure ratios, *RFR* resting full-cycle ratio, *VOCO* vessel-oriented composite outcome

The 2-year clinical follow-up was completed in 98.1% of the included patients, 1.9% were lost to follow-up. At 2 years, VOCO occurred in 19 lesions (5.0%). The Kaplan–Meier (lesion based) estimates for VOCO for RFR, dPR[entire] and dPR[WFP] are shown in Fig. 3, VOCO and its individual components are presented in Table 3. Vessels with NHPRs ≤ 0.89 were associated with a higher risk of VOCO than those with NHPRs > 0.89 (significant for RFR and dPR[WFP], non-significant trend for dPR[entire]). In a univariable model, HRs on a lesion level were 2.73 (95% CI 1.04–7.12, *p* = 0.041) for RFR ≤ 0.89, 1.91 (95% CI 0.62–5.86, *p* = 0.26) for dPR[entire] ≤ 0.89 and 2.70 (95% CI 1.00–7.28, *p* = 0.049) for dPR[WFP] ≤ 0.89 (Table 4, Supplemental Table 3a and 3b). In addition, diabetes mellitus, chronic kidney disease with a GFR < 30 ml/min/1.73 m^2^ and previous myocardial infarction were associated with a higher incidence of VOCO at 2 years (Table 4, Supplemental Table 3a and 3b). In a multivariable model, NHPRs ≤ 0.89, chronic kidney disease with a GFR < 30 ml/min/1.73 m^2^, previous myocardial infarction and diabetes showed similar HR for VOCO at 2 years without reaching statistical significance (Table 4, Supplemental Tables 3a and 3b).

**Table 3:** Cumulative incidence of clinical outcomes at 2 years according to non-hyperemic pressure ratios

	RFR > 0.89, *N* = 308	RFR ≤ 0.89, *N* = 69	*p* value	dPR[entire] > 0.89, *N* = 328	dPR[entire] ≤ 0.89, *N* = 49	*p* value	dPR[WFP] > 0.89, *N* = 319	dPR[WFP] ≤ 0.89, *N* = 58	*p* value
All-cause death	20 (6.5%)	5 (7.2%)	0.8	20 (6.1%)	5 (10%)	0.3	20 (6.3%)	5 (8.6%)	0.5
Cardiac death	3 (1.0%)	2 (2.9%)	0.2	3 (0.9%)	2 (4.1%)	0.07	3 (0.9%)	2 (3.4%)	0.1
Target vessel revascularization	9 (2.9%)	4 (5.8%)	0.2	11 (3.4%)	2 (4.1%)	0.7	9 (2.8%)	4 (6.9%)	0.1
Myocardial infarction in target vessel	4 (1.3%)	0 (0%)	0.4	4 (1.2%)	0 (0%)	0.5	4 (1.3%)	0 (0%)	0.4
VOCO*	12 (3.9%)	7 (10%)	0.03	15 (4.6%)	4 (8.2%)	0.2	13 (4.1%)	6 (10%)	0.04

Data are expressed as cumulative incidence of clinical outcomes and numbers of events. Cumulative incidence of events presented as Kaplan–Meier estimates during follow-up of 730 days. *p* values are log-rank in survival analysis for the comparison of cumulative incidence of events between negative and positive non-hyperemic pressure ratios

*dPR[entire]* diastolic pressure ratio during diastole, *dPR[WFP]* diastolic pressure ratio during wave-free period, RFR resting full-cycle ratio, VOCO* vessel-oriented composite outcomes (defined as a composite of cardiac death, target-vessel myocardial infarction, and ischemia-driven target lesion revascularization)

**Table 4:** Predictors of 2-year vessel-oriented composite outcome (VOCO) according to resting full-cycle ratio (RFR)

	Univariable Model			Multivariable Model	
		HR (95% CI)	*p* value	HR (95% CI)	*p* value
RFR ≤ 0.89		2.73 (1.04–7.12)	0.041	2.28 (0.81–6.40)	0.12
FFR groups (ref: FFR > 0.90)					
FFR 0.81–0.85		1.79 (0.51–6.34)	0.365		
FFR 0.86–0.90		0.62 (0.13–2.84)	0.534		
Age (per year increase)		1.03 (0.98–1.08)	0.235		
Female		0.98 (0.38–2.55)	0.970		
Hypertension		1.09 (0.34–3.47)	0.882		
Diabetes		2.68 (1.04–6.91)	0.042	2.33 (0.94–5.78)	0.067
Chronic kidney disease^a^		5.66 (1.86–17.3)	0.002	2.68 (0.72–9.91)	0.14
Target lesion of left anterior descending coronary artery		0.85 (0.36–2.00)	0.709		
Proximal location (vs mid/distal)		1.24 (0.48–3.19)	0.654		
Previous myocardial infarction		3.24 (1.22–8.59)	0.018	2.73 (0.92–8.08)	0.070
Lesion length (≥ 20 mm)		1.32 (0.42–4.20)	0.637		
Diameter stenosis (≥ 50%)		1.25 (0.52–3.01)	0.619		

^a^Estimated glomerular filtration rate (< 30 ml/min/1.73 m^2^)

*CI* confidence interval, *FFR* fractional flow reserve, *HR* hazard ratio, *RFR* resting full-cycle ratio, *VOCO* vessel-oriented composite outcomes (defined as a composite of cardiac death, target-vessel myocardial infarction, and ischemia-driven target lesion revascularization)

## Discussion 

We investigated the incremental prognostic information of NHPRs in a single-centre real-life cohort with deferred lesions based on FFR measurement. The principal findings of this study are as follows: RFR ≤ 0.89 and dPR[WFP] ≤ 0.89 were associated with an increased risk of VOCO in deferred lesions; dPR[entire] ≤ 0.89 showed a similar trend without reaching statistical significance. Common risk factors like diabetes mellitus, previous myocardial infarction and chronic kidney disease were associated with the 2-years VOCO rate.

Nowadays, the role of physiological assessment in decision-making for revascularization is well implemented in the guidelines [11]. Randomized trials have demonstrated that deferral of lesions with negative FFR reduces the rate of myocardial infarction [17], that percutaneous coronary intervention of lesions with positive FFR avoids the need for subsequent urgent revascularizations [2, 18] and that an FFR-based approach as compared to an angiography-based approach lowers the number of revascularizations and stent implantations with no excess in cardiovascular endpoints [19]. There is a continuous relationship between lower FFR values and the risk of clinical events [20, 21].

Data on NHPRs, which facilitate pressure recordings, since adenosine application is omitted, are continually expanding, with a focus on iFR, which is equivalent to dPR[WFP], i.e. the mean Pd/Pa during the wave-free period of diastole. In the prospective randomized DEFINE-FLAIR and iFR-SWEDEHEART trials, an iFR-guided approach was non-inferior to FFR-guiding in terms of 1-year and 5-year clinical outcomes [8–10], despite some discordance of iFR and FFR values leading to fewer revascularizations based on iFR. Several other NHPRs that assess various periods of the cardiac cycle have been developed due to the copyright protection of iFR and with the intention to further improve the validity of NHPRs. For example, RFR is propagated by a major manufacturer, frequently used and may have a hypothetical advantage over diastolic indices, as it assesses both the diastolic and systolic phase [5]. There are no randomized trials on NHPRs other than iFR, so strictly speaking non-inferiority of a NHPRs-based revascularization (except for an iFR-based) has not been proven, but all NHPRs are considered equivalent by many interventionalists due to a very high correlation [5, 6], which was confirmed by the present study.

The published literature regarding outcomes and non-iFR-NHPRs is based in particular on the combined populations of the 3V FFR-FRIENDS study and the ^13^N-ammonia PET registry [12–14, 22], which included Asian patients and had several exclusion criteria, e.g. left ventricular ejection fraction < 35% and chronic kidney disease, so the population was not representative for real-life patients in western countries. Our population had—besides ethnicity—relevant differences in baseline characteristics compared to the Asian population: our population was older (71.0 vs 63.8 years), had a higher proportion of female patients (35% vs 21%) and previous myocardial infarction (31% vs 8%) [12].

Like in the previous studies, NHPRs were calculated retrospectively and off-line in our study, so therapeutic decisions were based on FFR. We felt that a reasonable evaluation of the prognostic value of NHPRs should focus on patients, whose lesions were deferred based on a negative FFR. Otherwise, if revascularized patients were included in the analysis, outcome of patients with positive NHPRs would have been largely confounded by the impact of revascularization. We found that RFR and dPR[WFP] values ≤ 0.89 were associated with a higher risk for VOCO in deferred patients in Kaplan–Meier plots and a univariable model. This is in line with the 5-year outcome reported by Lee et al., who found a cumulative VOCO incidence at 5 years of 7.5% in patients with deferral and concordant negative indices and 14.4% in those with deferral and discordant indices [12]. In contrast, the report on the 2-year outcome in the same Asian population found no increased risk of VOCO in patients with discordant indices, but only in cases with concordant positive values [13], the reason for this discrepancy remains unclear. A very recently published multi-centre registry confirmed a prognostic impact of non-hyperemic Pd/Pa on top of FFR [23]﻿. However, this study did not include RFR or iFR.

For interventionalists, it is particularly important to confirm the safety of deferral based on NHPRs values > 0.89 [13, 14], as many catheter laboratories use NHPRs alone. In other words, do we need FFR in lesions considered for deferral based on NHPRs? This question cannot be answered reliably by our study, since the decision to revascularize or defer was based on FFR. Our study confirmed previous findings of a very high correlation of iFR and other NHPRs, thus it appears reasonable that the non-inferiority of iFR demonstrated by two outcome trials can be conferred to other NHPRs [8–10]. In our whole cohort including revascularized and deferred patients, there were more patients with positive RFR/negative FFR than vice versa (13.2% vs 8.8%) [6], i.e. revascularization would have been performed slightly more often based on NHPRs than it actually was based on FFR, and discordance with positive FFR/negative NHPRs was infrequent. Thus, only few patients would have been deferred based on NHPRs, while they would have been revascularized based on FFR, so the risk of deferral based on NHPRs appears to be low in real-life clinical practice. This contrasts with the reports of Lee et al. and the landmark iFR trials, where the revascularization rate was lower with a NHPRs-based strategy. One explanation may be the higher age and higher proportion of females in our population, since young age and male gender are known to be associated with negative NHPRs/positive FFR discordance [13]. RFR suggested the presence of flow-limiting stenoses in more patients than dPR[WFP] and dPR[entire], which is consistent with previous studies [13, 14] and will further decrease the risk of deferral, if RFR is used.

The overall rate of discordant indices was higher in our study than in the study by Lee et al. (13–18% vs 11–13%), so discordance is a frequent phenomenon in clinical routine, although it does not always become evident, since recordings of both FFR plus NHPRs are not always performed [13]. This underlines the importance of one crucial question: “Should we revascularize patients with discordant indices?” Although current evidence demonstrates the increased cardiovascular risk of patients with discordant indices, and one may draw the conclusion that these patients are no appropriate candidates for deferral, available data do not really help in answering this question, since the decision to revascularize was never based on the presence of discordance of indices. In the light of the ongoing debate on the prognostic benefit of percutaneous coronary revascularization as well as FFR-guidance in general, we consider it improbable that this question will be ever answered. Thus, while lesions with concordant negative indices can be safely and should be deferred, those with discordant indices may be considered for revascularization. Surely, due to their high cardiovascular risk, patients with discordant indices should be especially encouraged to use all preventive measures to lower cardiovascular risk.

As compared to RFR and dPR[WFP], dPR[entire] was more often negative. While outcome was significantly worse for lesions with RFR or dPR[WFP] ≤ 0.89, there was only a non-significant trend towards an increased risk for dPR[entire] ≤ 0.89 and the hazard ratio was smaller. This discrepancy between NHPRs may point to a varying reliability in risk prediction, but may also be explained by the low statistical power of our single-centre study, since the association of NHPRs with the estimated risk of VOCO was uniform for all NHPRs in a previous study [12].

### Study limitations

This study has important limitations related to its single-centre, observational design and should be considered as hypothesis-generating. Endpoints were collected by telephone follow-up and evaluation of available clinical records, so the probability of missed or misclassified endpoints must be considered higher than in prospectively designed trials. Since our population included many older and sicker patients, VOCO comprised a relevant number of deaths, whose causal relationship to the deferred lesion mostly remained unclear. Diagnoses of myocardial infarction were derived from medical reports, and we could not verify, if diagnoses complied exactly with applicable definitions of myocardial infarction.

Our main finding, i.e. the prognostic implication of NHPRs on top of FFR, was supported by Kaplan–Meier curves/log-rank test and HRs in the univariable analysis, but statistical significance was not reached for HRs in the multivariable analysis. The number of events was small, which probably impeded significance levels in the multivariable analysis, especially since HRs remained high, but 95% CIs were wide, and well-known risk factors like chronic kidney disease and diabetes lost statistical significance in the multivariable model, too. Moreover, our findings are supported by the above-mentioned multi-centre registry, which demonstrated a prognostic impact of non-hyperemic Pd/Pa on top of FFR in a multivariate cox-regression analysis [23].

Our cohort had few exclusion criteria, so the population was heterogenous. We consider this broad “all-comers” population as strength, since it probably better reflects patients undergoing coronary angiography in real life than previous studies with more extensive exclusion criteria.

## Conclusion

In a large cohort representative of real-life practice, deferred lesions with positive RFR and dPR[WFP] despite a negative FFR were associated with a higher incidence for VOCO at 2 years, suggesting that the use of NHPRs in addition to FFR may improve risk estimation. RFR and dPR[WFP] had an excellent correlation and equal accuracy. Although these results should be interpreted with caution due to the study design and low number of events, our study supports the use of NHPRs to facilitate implementation of pressure wire recordings in the catheter laboratory.

## Supplementary Information

Below is the link to the electronic supplementary material.

Supplementary file1 (DOCX 40 KB)

## Data Availability

The datasets generated during and/or analysed during the current study are available from the corresponding author on reasonable request.

## Abbreviations

CI: Confidence interval
dPR[entire]: Diastolic pressure ratio during entire diastole
dPR[WFP]: Diastolic pressure ratio during the wave-free period of the diastole
FFR: Fractional flow reserve
HR: Hazard ratio
iFR: Instantaneous wave-free ratio
IQR: Interquartile range
NHPRs: Non-hyperemic pressure ratios
Pa: Proximal aortic pressure
Pd: Distal arterial pressure
RFR: Resting full-cycle ratio
VOCO: Vessel-oriented composite outcome
