# Supplementary material for: Prognostic impact of resting full-cycle ratio and diastolic non-hyperemic pressure ratios in patients with deferred revascularization
Source: Clin Res Cardiol. 2023 Jan 5;112(9):1220–30. doi: 10.1007/s00392-022-02149-1 (PMC10449998; doi:10.1007/s00392-022-02149-1)
Supplement: Supplementary file 1 — Supplementary file1 (DOCX 40 KB) [file 392_2022_2149_MOESM1_ESM.docx]

**SUPPLEMENTAL MATERIAL**

| **Table 1a. Baseline characteristics according to diastolic pressure ratio during entire diastole (dPR[entire])** | | | | |
| --- | --- | --- | --- | --- |
|  |  | dPR[entire]>0.89,  N=274 | dPR[entire]≤0.89,  N=42 | p-value |
| **Female** |  | 96.0 (35.0%) | 14.0 (33.3%) | 0.83 |
| **Age (years)** |  | 70.5 (62.0-77.0) | 75.0 (67.2-81.0) | 0.005 |
| **Body-mass-index (kg/m^2^)** |  | 27.1 (24.4-30.5) | 27.4 (23.9-30.5) | 0.73 |
| **Diabetes** |  | 73.0 (26.6%) | 9.0 (21.4%) | 0.47 |
| **Hypertension** |  | 195.0 (71.2%) | 31.0 (73.8%) | 0.72 |
| **Dyslipidemia** |  | 133.0 (48.5%) | 23.0 (54.8%) | 0.45 |
| **Atrial fibrillation** |  | 35.0 (12.8%) | 7.0 (16.7%) | 0.49 |
| **Former or current smoker** |  | 85.0 (31.0%) | 17.0 (40.5%) | 0.22 |
| **Peripheral artery disease** |  | 17.0 (6.2%) | 6.0 (14.3%) | 0.10 |
| **Previous stroke** |  | 26.0 (9.5%) | 8.0 (19.0%) | 0.10 |
| **Family history of coronary artery disease** |  | 33.0 (12.0%) | 7.0 (16.7%) | 0.40 |
| **eGFR (ml/min/1.73 m^2^)** |  | 63.0 (48.5-79.9) | 50.9 (42.9-73.0) | 0.12 |
| **Chronic kidney disease*** |  | 16.0 (5.8%) | 4.0 (9.5%) | 0.32 |
| **Previous coronary artery bypass graft** |  | 20.0 (7.3%) | 3.0 (7.1%) | >0.99 |
| **Previous myocardial infarction** |  | 80.0 (29.2%) | 17.0 (40.5%) | 0.14 |
| **Clinical presentation** |  |  |  | 0.90 |
| Acute coronary syndrome |  | 50.0 (18.2%) | 8.0 (19.0%) |  |
| Stable coronary artery disease |  | 224.0 (81.8%) | 34.0 (81.0%) |  |
| **Lipid-lowering drugs** |  | 248.0 (90.5%) | 38.0 (90.5%) | >0.99 |
| Values are expressed as median (IQR) or n (%). eGFR = estimated glomerular filtration rate; *estimated glomerular filtration rate (<30 ml/min/1.73 m^2^) | | | | |

| **Table 2a. Lesion characteristics according to diastolic pressure ratio during entire diastole (dPR[entire])** | | | | |
| --- | --- | --- | --- | --- |
|  |  | dPR[entire]>0.89,  N=328 | dPR[entire]≤0.89,  N=49 | p-value |
| **Location of lesions** |  |  |  |  |
| Left anterior descending artery |  | 151 (46.0%) | 43 (87.8%) | <0.001 |
| Circumflex artery |  | 75 (22.9%) | 2 (4.1%) | 0.002 |
| Right coronary artery |  | 5 (1.5%) | 1 (2.0%) | 0.57 |
| Ramus intermedius |  | 97 (29.6%) | 3 (6.1%) | <0.001 |
| **Proximal lesion** |  | 154 (47.0%) | 20 (40.8%) | 0.42 |
| **Reference diameter (mm)** |  | 3.03 (2.69-3.44) | 2.82 (2.45-3.17) | 0.011 |
| **Minimum lumen diameter (mm)** |  | 1.53 (1.31-1.81) | 1.35 (1.16-1.58) | 0.002 |
| **Diameter stenosis (%)** |  | 50.0 (47.0-53.0) | 53.0 (50.0-55.0) | 0.019 |
| **Lesion length (mm)** |  | 11.78 (8.39-17.50) | 11.06 (7.39-18.18) | 0.64 |
| **Fractional flow reserve** |  | 0.88 (0.86-0.92) | 0.84 (0.83-0.86) | <0.001 |
| **Resting full-cycle ratio** |  | 0.95 (0.92-0.98) | 0.87 (0.86-0.88) | <0.001 |
| **dPR[entire]** |  | 0.95 (0.93-0.98) | 0.88 (0.87-0.89) | <0.001 |
| **dPR[WFP]** |  | 0.95 (0.93-0.98) | 0.87 (0.86-0.89) | <0.001 |
| Values are expressed as median (IQR) or n (%). dPR[entire] = diastolic pressure ratio during entire diastole, dPR[WFP] = diastolic pressure ratio during the wave-free period of the diastole | | | | |

| **Table 3a. Predictors of 2-Year vessel-oriented composite outcome (VOCO) according to diastolic pressure ratio during entire diastole (dPR[entire])** | | | | | |
| --- | --- | --- | --- | --- | --- |
|  | Univariable Model | | | Multivariable Model | |
|  |  | HR (95% CI) | p-value | HR (95% CI) | p-value |
| **dPR[entire]**≤**0.89** |  | 1.91 (0.62-5.86) | 0.26 | 1.86 (0.53-6.52) | 0.33 |
| **FFR groups (ref:** *FFR>0.90)* |  |  |  |  |  |
| *FFR 0.81-0.85* |  | 1.84 (0.58-5.80) | 0.30 |  |  |
| *FFR 0.86-0.90* |  | 0.63 (0.15-2.62) | 0.52 |  |  |
| **Age (per year increase)** |  | 1.03 (0.98-1.08) | 0.24 |  |  |
| **Female** |  | 0.98 (0.38-2.55) | 0.97 |  |  |
| **Hypertension** |  | 1.09 (0.34-3.47) | 0.88 |  |  |
| **Diabetes** |  | 2.68 (1.04-6.91) | 0.042 | 2.33 (0.88-6.20) | 0.090 |
| **Chronic kidney disease*** |  | 5.66 (1.86-17.3) | 0.002 | 3.08 (0.91-10.5) | 0.071 |
| **Target lesion of left anterior descending coronary artery** |  | 0.85 (0.36-2.00) | 0.71 |  |  |
| **Proximal location (vs mid/distal)** |  | 1.24 (0.48-3.19) | 0.65 |  |  |
| **Previous myocardial infarction** |  | 3.24 (1.22-8.59) | 0.018 | 2.79 (0.96-8.11) | 0.060 |
| **Lesion length (≥ 20mm)** |  | 1.32 (0.42-4.20) | 0.64 |  |  |
| **Diameter stenosis (≥ 50%)** |  | 1.25 (0.52-3.01) | 0.62 |  |  |
| *estimated glomerular filtration rate (<30 ml/min/1.73 m^2^), CI = confidence interval, dPR[entire] = diastolic pressure ratio during entire diastole, FFR = Fractional flow reserve, HR = hazard ratio, VOCO = vessel-oriented composite outcomes (defined as a composite of cardiac death, target-vessel myocardial infarction, and ischemia-driven target lesion revascularization) | | | | | |

| **Table 1b. Baseline characteristics according to diastolic pressure ratio during the wave-free period of the diastole (dPR[WFP])** | | | | |
| --- | --- | --- | --- | --- |
|  |  | dPR[WFP]> 0.89,  N=266 | dPR[WFP]≤ 0.89,  N=50 | p-value |
| **Female** |  | 92 (34.6%) | 18 (36.0%) | 0.85 |
| **Age (years)** |  | 70.0 (62.0-77.0) | 76.0 (68.0-81.0) | <0.001 |
| **Body-mass-index (kg/m^2^)** |  | 27.4 (24.5-30.5) | 26.3 (23.4-29.3) | 0.10 |
| **Diabetes** |  | 71 (26.7%) | 11 (22.0%) | 0.49 |
| **Hypertension** |  | 190 (71.4%) | 36 (72.0%) | 0.93 |
| **Dyslipidemia** |  | 129 (48.5%) | 27 (54.0%) | 0.48 |
| **Atrial fibrillation** |  | 34 (12.8%) | 8 (16.0%) | 0.54 |
| **Former or current smoker** |  | 83 (31.2%) | 19 (38.0%) | 0.35 |
| **Peripheral artery disease** |  | 15 (5.6%) | 8 (16.0%) | 0.017 |
| **Previous stroke** |  | 25 (9.4%) | 9 (18.0%) | 0.072 |
| **Family history of coronary artery disease** |  | 32 (12.0%) | 8 (16.0%) | 0.44 |
| **eGFR (ml/min/1.73 m^2^)** |  | 63.2 (49.0-81.2) | 50.5 (38.2-70.6) | 0.006 |
| **Chronic kidney disease*** |  | 15 (5.6%) | 5 (10.0%) | 0.34 |
| **Previous coronary artery bypass graft** |  | 20 (7.5%) | 3 (6.0%) | >0.99 |
| **Previous myocardial infarction** |  | 79 (29.7%) | 18 (36.0%) | 0.38 |
| **Clinical presentation** |  |  |  | 0.64 |
| Acute coronary syndrome |  | 50 (18.8%) | 8 (16.0%) |  |
| Stable coronary artery disease |  | 216 (81.2%) | 42 (84.0%) |  |
| **Lipid-lowering drugs** |  | 242 (91.0%) | 44 (88.0%) | 0.60 |
| Values are expressed as median (IQR) or n (%). eGFR, estimated glomerular filtration rate; *estimated glomerular filtration rate (<30 ml/min/1.73 m^2^) | | | | |

| **Table 2b. Lesion characteristics according to diastolic pressure ratio during the wave-free period of the diastole (dPR[WFP])** | | | | |
| --- | --- | --- | --- | --- |
|  |  | dPR[WFP]> 0.89,  N=319 | dPR[WFP]≤ 0.89,  N=58 | p-value |
| **Location of lesions** |  |  |  |  |
| Left anterior descending artery |  | 146 (45.8%) | 48 (82.8%) | <0.001 |
| Circumflex artery |  | 73 (22.9%) | 4 (6.9%) | 0.005 |
| Right coronary artery |  | 5 (1.6%) | 1 (1.7%) | >0.99 |
| Ramus intermedius |  | 95 (29.8%) | 5 (8.6%) | <0.001 |
| **Proximal lesion** |  | 150 (47.0%) | 24 (41.4%) | 0.43 |
| **Reference diameter (mm)** |  | 3.03 (2.69-3.44) | 2.87 (2.46-3.20) | 0.018 |
| **Minimum lumen diameter (mm)** |  | 1.53 (1.31-1.81) | 1.40 (1.16-1.60) | 0.002 |
| **Diameter stenosis (%)** |  | 50.0 (47.0-53.0) | 52.5 (49.25-54.75) | 0.016 |
| **Lesion length (mm)** |  | 11.75 (8.34-17.76) | 11.13 (7.56-16.85) | 0.67 |
| **Fractional flow reserve** |  | 0.89 (0.86-0.93) | 0.84 (0.83-0.86) | <0.001 |
| **Resting full-cycle ratio** |  | 0.95 (0.92-0.98) | 0.87 (0.86-0.88) | <0.001 |
| **dPR[entire]** |  | 0.96 (0.93-0.98) | 0.89 (0.87-0.89) | <0.001 |
| **dPR[WFP]** |  | 0.95 (0.93-0.98) | 0.88 (0.86-0.89) | <0.001 |
| Values are expressed as median (IQR) or n (%). dPR[entire] = diastolic pressure ratio during entire diastole, dPR[WFP] = diastolic pressure ratio during the wave-free period of the diastole | | | | |

| **Table 3b. Predictors of 2-Year vessel-oriented composite outcome (VOCO) according to diastolic pressure ratio during the wave-free period of the diastole (dPR[WFP])** | | | | | |
| --- | --- | --- | --- | --- | --- |
|  | Univariable Model | | | Multivariable Model | |
|  |  | HR (95% CI)^1^ | p-value | HR (95% CI)^1^ | p-value |
| **dPR[WFP]≤0.89** |  | 2.70 (1.00-7.28) | 0.049 | 2.38 (0.81-6.94) | 0.11 |
| **FFR groups (ref:** *FFR>0.90)* |  |  |  |  |  |
| *FFR 0.81-0.85* |  | 1.79 (0.51-6.34) | 0.365 |  |  |
| *FFR 0.86-0.90* |  | 0.62 (0.13-2.84) | 0.534 |  |  |
| **Age (per year increase)** |  | 1.03 (0.98-1.08) | 0.235 |  |  |
| **Female** |  | 0.98 (0.38-2.55) | 0.970 |  |  |
| **Hypertension** |  | 1.09 (0.34-3.47) | 0.882 |  |  |
| **Diabetes** |  | 2.68 (1.04-6.91) | 0.042 | 2.38 (0.95-5.91) | 0.063 |
| **Chronic kidney disease*** |  | 5.66 (1.86-17.3) | 0.002 | 2.74 (0.76-9.93) | 0.12 |
| **Target lesion of left anterior descending coronary artery** |  | 0.85 (0.36-2.00) | 0.709 |  |  |
| **Proximal location (vs mid/distal)** |  | 1.24 (0.48-3.19) | 0.654 |  |  |
| **Previous myocardial infarction** |  | 3.24 (1.22-8.59) | 0.018 | 2.79 (0.95-8.16) | 0.061 |
| **Lesion length (≥ 20mm)** |  | 1.32 (0.42-4.20) | 0.637 |  |  |
| **Diameter stenosis (≥ 50%)** |  | 1.25 (0.52-3.01) | 0.619 |  |  |
| *estimated glomerular filtration rate (<30 ml/min/1.73 m^2^), CI = confidence interval, dPR[WFP] = diastolic pressure ratio during the wave-free period of the diastole, FFR = fractional flow reserve, HR = hazard ratio, VOCO = vessel-oriented composite outcomes (defined as a composite of cardiac death, target-vessel myocardial infarction, and ischemia-driven target lesion revascularization) | | | | | |
